# Risk Estimation of HNA-3 Incompatibility and Alloimmunization in Thai Populations

**DOI:** 10.1371/journal.pone.0116905

**Published:** 2015-01-21

**Authors:** Oytip Nathalang, Kamphon Intharanut, Kanokpol Siriphanthong, Siriporn Nathalang, Nipapan Leetrakool

**Affiliations:** 1 Graduate Program in Biomedical Sciences, Faculty of Allied Health Sciences, Thammasat University, Pathumtani, Thailand; 2 National Blood Centre, Thai Red Cross Society, Bangkok, Thailand; 3 Blood Bank Section, Faculty of Medicine, Chiang Mai University, Chiang Mai, Thailand; University of Cape Town, SOUTH AFRICA

## Abstract

Severe transfusion-related acute lung injury (TRALI) is often due to antibodies in blood components directed against human neutrophil antigen (HNA)-3a. This study aimed to report the genotype frequencies of the HNA-3 system and to estimate the potential risk of HNA-3 incompatibility and alloimmunization in two Thai populations. Eight hundred DNA samples obtained from 500 unrelated healthy blood donors at the National Blood Centre, Thai Red Cross Society, Bangkok and 300 samples from the Blood Bank, Faculty of Medicine, Chiang Mai University, Chiang Mai, Thailand were included. HNA-3 genotyping was performed using an in-house polymerase chain reaction with sequence-specific primer (PCR-SSP) technique. The observed frequencies of the HNA-3a/3a, HNA-3a/3b, and HNA-3b/3b genotypes were 0.528, 0.380, and 0.092 in central Thais and 0.600, 0.350, and 0.050 in northern Thais, respectively. The frequencies were used to estimate HNA-3 incompatibility and risk of HNA-3a alloimmunization. The HNA-3 incompatibility in central Thais (33.28%) was higher than northern Thais (28.75%), corresponding to a significantly higher probability of HNA-3a alloimmunization (P<0.05) similar to Japanese and Chinese populations. This study showed the high risk of HNA-3 incompatibility and alloimmunization, especially in central Thai blood donors. A molecular-based identification of the HNA-3 genotype of female donors is suggested to reduce the risk of TRALI following plasma and whole blood allogeneic transfusion.

## Introduction

Alloantibodies against human neutrophil alloantigens (HNA) can be formed during pregnancy or transfusion of blood components and are associated with neonatal alloimmune neutropenia, febrile transfusion reactions and transfusion-related acute lung injury (TRALI) [1]. Among HNA alloantibodies, the HNA-3a antibodies in donor plasma directed against recipient WBC antigens are frequently implicated in severe and fatal TRALI [2–4]. For Asian populations, large scale screenings of HNA-3a antibodies in blood donors are unprofitable and unavailable. Therefore, only HNA genotyping is implemented for population studies and the distribution of HNA gene frequencies showed significant differences between Caucasian and non Caucasian populations [5–11].

The HNA-3 antigens are associated with a biallelic polymorphism caused by a single nucleotide polymorphism, SNP (c.461G>A; p.Arg154Gln) in the choline transporter like protein-2 (CTL-2, Gene *SLC44A2*) [3,4]. This finding leads to DNA-based HNA-3 genotyping by polymerase chain reaction with sequence-specific primer (PCR-SSP), widely used to determine genotypes as HNA-3a/3a, HNA-3a/3b, and HNA-3b/3b, respectively [12]. Importantly, an additional SNP (c.457C>T, p.Leu153Phe) was previously reported that may yield false negative genotyping results for HNA-3a, especially in the HNA-3a/3b genotype, if the specific primer encompasses the mutation [13]. The SNP c.457C>T mutation can be found in German and African American blood donors [13,14]; while, it has not been found in Japanese and Chinese populations [8,9]. A recent study in Thai blood donors reported that the gene frequencies of *HNA-3a* and *HNA-3b* were 0.490 and 0.510, respectively and HNA-3a/3b individuals were the most common; however, the SNP c.457C>T mutation was not determined [11].

HNA-3 genotyping may be applied to screen for apheresis donors capable of developing anti-HNA-3a antibodies, especially in pregnant women who are homozygous for HNA-3b. To avoid misreading interpretations of HNA-3b/3b, screening for the SNP c.457C>T mutation must be performed together with HNA-3 genotyping by PCR-SSP. These will be helpful to correctly predict the risk of HNA-3 alloimmunization. The purpose of this study was to report the genotype frequencies of the HNA-3 system and to estimate the potential risk of HNA-3 incompatibility and alloimmunization in Thai populations.

## Materials and Methods

### Samples

Peripheral venous blood was collected in EDTA-anticoagulated blood from 800 unrelated healthy Thai blood donors. Five hundred samples were from the National Blood Centre, Thai Red Cross Society, Bangkok and 300 samples were from the Blood Bank, Faculty of Medicine, Chiang Mai University, Chiang Mai, Thailand. Written informed consent was obtained from each subject. This study was approved by the Committee on Human Rights Related to Research Involving Human Subjects, Thammasat University, Pathumtani, Thailand. Genomic DNA was extracted from peripheral blood samples using a Genomic DNA extraction kit (REAL Genomics, RBCBioscience, Taipei, Taiwan), and the salting out method [15] and was then stored at −20°C until used for genotyping.

### DNA standards

Known HNA-3a and -3b DNA samples were provided by Dr. Núria Nogués, Laboratori d’Immunohematologia, Banc de Sang i Teixits, Passeig Taulat, Barcelona, Spain. In addition, a DNA control for the *SLC44A2* gene with the c.457T was synthesized using the GeneArt, Strings technology (Life Technologies, Invitrogen, Singapore) and served as PCR template.

### HNA-3 genotyping by PCR-SSP

HNA-3 genotyping was performed by a PCR-SSP technique, as previously described [12] with some modifications. Briefly, 1 μL of genomic DNA (50 ng/μL) was amplified in a total volume of 10 μL using 10 μM of HNA-3ab reverse primer 5’-GTGCGCCAATATCCTCACTTG-3’, 1 μL and 10 μM of HNA-3a forward primer 5’-AGTGGCTGAGGTGCTTCG-3’, 1 μL for *HNA-3a* genotyping and 10 μM of HNA-3ab reverse primer, 1 μL and 10 μM of HNA-3b forward primers 5’-GAGTGGCTGAGGTGCTTCA-3’, 1 μL for *HNA-3b* genotyping. The human growth hormone (*HGH*) gene was co-amplified as internal control using 10 μM HGH forward primer 5’-TGCCTTCCCAACCATTCCCTTA-3’, 1 μL, and 10 μM HGH reverse primer 5’-CCACTCACGGATTTCTGTTGTGTTTC-3’, 1 μL. The PCR was performed with 5 μL of PCR reaction mixture (OnePCR Plus, GeneDirex, Taiwan) in a G-STORM GS1 thermal cycler (Gene Technologies Ltd., Somerset, UK).

PCR was performed under the conditions described below, e.g., 95°C for 10 min (initial denaturation). The cycle parameters of the PCR program began with the first step of 10 cycles of 30 sec at 95°C, 40 sec at 64°C and 30 sec at 72°C, then 20 cycles of 30 sec at 95°C and 30 sec at 61°C and 30 sec at 72°C. The last step was final extension 5 min at 72°C and the sample was kept at 4°C.

PCR products were resolved by 1.5% agarose gel electrophoresis at 100 volts in 1X TBE buffer and were visualized under blue-light transilluminator. The PCR product size of the *HNA-3a* and *-3b* alleles was 291 bp, whereas that of the internal control the *HGH* gene was 434 bp.

### Typing of SNP c.457C>T by PCR-SSP

Typing of SNP c.457C>T was performed by a PCR-SSP technique, as previously described [13] with some modifications. Briefly, 1 μL of genomic DNA (50 ng/μL) was amplified in a total volume of 10 μL using 10 μM of HNA-3var reverse primer 5’-CATGCCCATCCTCATAGGTC-3’, 1 μL and 10 μM of HNA-3var-C forward primer 5’-CAGGGAGTGGCTGAGGTGC-3’, 1 μL for SNP c.457C typing and 10 μM of HNA-3var reverse primer, 1 μL and 10 μM of HNA-3var-T forward primers 5’-CAGGGAGTGGCTGAGGTGT-3’, 1 μL for SNP c.457T typing. The *HGH* gene was co-amplified as internal control as described in the previous section. The PCR was performed with 5 μL of PCR reaction mixture (OnePCR Plus, GeneDirex, Taiwan) in a G-STORM GS1 thermal cycler (Gene Technologies Ltd., Somerset, UK).

PCR conditions were similar to HNA-3 genotyping except the first step of the PCR program was changed to 10 cycles of 30 sec at 95°C, 40 sec at 68°C and 30 sec at 72°C and PCR product size of both SNPs c.457C/T was 238 bp.

### DNA sequencing

Genomic DNA of 19 genotyped blood donors (four HNA-3a/3a, 13 HNA-3a/3b, and two HNA-3b/3b) was sequenced to confirm the result of PCR-SSP. A fragment of 663 bp containing both SNPs (c.461G>A and c.457C>T) was obtained from PCR amplification of genomic DNA using the forward primer 5’- TTTTTCCTCTCCCCGCTTCC-3’ and reverse primer 5’-ATGATCAGGCCAACCACAGG-3’ and using similar PCR conditions to HNA-3 genotyping.

### Statistical analysis

Gene frequencies were calculated by gene-counting method. The Chi-square (X^2^) test was used to evaluate whether the observed genotype frequencies were in agreement with the expected ones under the Hardy-Weinberg equilibrium. The frequencies of HNA-3 incompatibility were estimated, based on the HNA-3 gene frequencies of each population. The percentage of HNA-3 incompatibilities was calculated by [3a/3a(3b/3b+3a/3b) + 3b/3b(3a/3a+3a/3b)]; 3a/3a, 3a/3b, and 3b/3b were the percentage of genotype frequencies in each population. In addition, the estimation risk of HNA-3a alloimmunization was obtained by multiplying the probability of being predicted HNA-3a negative phenotype frequency by the probability of having a predicted HNA-3a phenotype frequency. The Chi-square test of homogeneity was used to determine the difference between central Thai, northern Thai and other populations. A P value equal or less than 0.05 was considered statistically significant.

## Results

DNA from 800 blood donors was collected and genotyped for HNA-3 by PCR-SSP. The determined HNA-3 genotypes among 500 central Thais and 300 northern Thais were consistent with the Hardy-Weinberg equilibrium (P = 0.952 and P = 0.997). In central Thais, the genotype frequencies of HNA-3a/3a, HNA3a/3b, and HNA-3b/3b were 0.528(264/500), 0.380(190/500), and 0.092(46/500), respectively, while they were 0.600(180/300), 0.350(105/300), and 0.050(15/300) in northern Thais, respectively. The comparison of the HNA-3 genotypes between central and northern Thais revealed that the frequency of the HNA-3a genotype was significantly higher in northern Thais (P = 0.014), as shown in Table 1.

**Table 1:** Comparison of observed and expected gene frequencies in central and northern Thai populations.

**HNA genotype**	**Central Thais (n = 500)**			**Northern Thais (n = 300)**			**Homogeneity test**
	**Observed frequency**	**Expected frequency**	**Hardy-Weinberg analysis**	**Observed frequency**	**Expected frequency**	**Hardy-Weinberg analysis**	
**HNA3a/3a**	0.528	0.515	X^2^ = 0.0037 P = 0.952	0.600	0.600	X^2^ = 0.0000 P = 0.997	X^2^ = 6.0300 P = 0.014
**HNA3a/3b**	0.380	0.405		0.350	0.349		
**HNA3b/3b**	0.092	0.080		0.050	0.051		

In this study, the SNP c.457C>T variation was investigated by PCR-SSP in all samples and no cases of c.457T were found. To validate the PCR-SSP technique, 19 samples were sequenced to SNPs c.461G>A and c.457C>T and the results showed c.461G in all HNA-3a individuals, c.461A in all HNA-3b individuals and c.461G/A in all HNA-3a/3b individuals, while none of the 19 samples carried SNP c.457T.

An estimation of the HNA-3 incompatibilities in the two investigated Thai populations showed values of 33.28% (central Thais) and 28.75% (northern Thais). The risk of HNA-3a alloimmunization in central and northern Thais was significantly different (P<0.05) at 0.0835 and 0.0475, respectively. Compared with previous studies among other populations, the risk of HNA-3a alloimmunization in central Thais was similar to Asian populations, but was significantly different (P<0.05) from English Caucasian, Danish, and Zambians, as shown in Table 2.

**Table 2:** Estimations of HNA-3 incompatibilities and risk of HNA-3a alloimmunization among populations.

**Population**	**Number**	**HNA-3 incompatibilities (%)**	**The frequencies of predicted HNA-3a phenotype**		**Risk of HNA-3a alloimmunization**
			**Negative**	**Positive**	
**Central Thais**	500	33.28	0.092	0.908	0.0835
**Northern Thais**	300	28.75	0.050	0.950	0.0475^a^
**English Caucasian**[5]	140	27.36	0.029	0.971	0.0282^a^
**German**[6]	119	30.95	0.067	0.933	0.0625
**Turkish**[6]	118	32.43	0.085	0.915	0.0778
**Danish**[7]	366	26.06	0.041	0.959	0.0393^a^
**Zambians**[7]	193	4.91	0.000	1.000	0.000^a^
**Japanese**[8]	570	34.15	0.111	0.889	0.0987
**Zhejiang Han Chinese**[9]	400	36.13	0.132	0.868	0.1146
**Guangzhou Han Chinese**[10]	195	30.22	0.056	0.944	0.0529

^a^Significant differences from central Thais (P<0.05; Chi-square test)

## Discussion

HNA-3a alloantibodies found in donor plasma directed against recipient’s neutrophils can cause severe and fatal TRALI [2–4]. However, in Thailand an implementation of HNA-3a antibody screening using serological tests has not been feasible for routine testing in blood donors because commercial kits are expensive and development of an in-house test kit requires both specific antisera and HNA panels. A previous study in Thai blood donors reported that HNA-3 genotypes could be determined by PCR-SSP technique [11]. However, the additional analysis of the non-synonymous SNP c.457C>T is required to determine the corrected HNA-3a genotype.

This study is the first report comparing the genotype frequencies of HNA-3 between central and northern Thai populations including analysis of SNP c.457C>T. Blood samples of 500 central Thai blood donors and 300 northern Thai blood donors were genotyped for the HNA-3 system by an in-house PCR-SSP. The observed higher prevalence of HNA-3b/3b in the samples from central Thais, 9.2% versus 5.0% in northern Thais demonstrates the different risks of anti-HNA-3a production in the two populations. However, to exclude the possibility of mistyping for HNA-3a determination, all samples were tested for the SNP c.457T variation by PCR-SSP and we found no cases of this variation, similar to Japanese, Chinese and Brazilian populations [8,9,16]. Also, the determined HNA-3 genotypes by PCR-SSP were randomly confirmed by DNA sequencing and the results showed 100% concordance.

Genotype frequencies of the HNA-3 system obtained by PCR-SSP were also used to calculate the probabilities of HNA-3 incompatibility and alloimmunization risks. It was found that the probability of HNA-3 incompatibility in central Thais was higher than in northern Thais resulting in a significantly increased risk of HNA-3a alloimmunization in HNA-3b/3b individuals from central Thais. The exposure to the HNA-3a antigen may originate from incompatible blood transfusion or from pregnancy and the observed increased risk in central Thais is similar to that previously reported in Japanese and Han Chinese [8,9]. To reduce the risk of TRALI, the exclusion of female donors with a history of pregnancy from plasma preparation was introduced by the National Blood Service, UK in 2003. In a subsequent analysis of the annual number of TRALI cases reported to the Serious Hazards of Transfusion (SHOT) Scheme was found decreased. Additionally, screening apheresis donors for human leukocyte antigen (HLA) and HNA antibodies was suggested [17]. Due to the equal ratio of male and female donors in Thailand, it is not practical to exclude female donors from plasma preparation. We suggest that identifying the HNA-3b/3b genotype in female blood donors is better than screening for HLA and HNA antibodies in apheresis donors to minimize the TRALI adverse events.

In conclusion, it is clear from this study that the risk of HNA-3a alloimmunization in central Thais seems high. A molecular-based identification of the HNA-3 genotypes of female donor screening should facilitate a lower the risk of TRALI following plasma and whole blood allogeneic transfusion.
